# Quantification of Beta Adrenergic Receptor Subtypes in Beta-Arrestin Knockout Mouse Airways

**DOI:** 10.1371/journal.pone.0116458

**Published:** 2015-02-06

**Authors:** Akhil Hegde, Ryan T. Strachan, Julia K. L. Walker

**Affiliations:** 1 Division of Pulmonary, Allergy and Critical Care Medicine, Duke University Medical Center, Durham, North Carolina, United States of America; 2 Howard Hughes Medical Institute and Department of Medicine, Duke University Medical Center, Durham, North Carolina, United States of America; University of North Dakota, UNITED STATES

## Abstract

In allergic asthma Beta 2 adrenergic receptors (β_2_ARs) are important mediators of bronchorelaxation and, paradoxically, asthma development. This contradiction is likely due to the activation of dual signaling pathways that are downstream of G proteins or β-arrestins. Our group has recently shown that β-arrestin-2 acts in its classical role to desensitize and constrain β_2_AR-induced relaxation of both human and murine airway smooth muscle. To assess the role of β-arrestins in regulating β_2_AR function in asthma, we and others have utilized β-arrestin-1 and -2 knockout mice. However, it is unknown if genetic deletion of β-arrestins in these mice influences β_2_AR expression in the airways. Furthermore, there is lack of data on compensatory expression of βAR subtypes when either of the β-arrestins is genetically deleted, thus necessitating a detailed βAR subtype expression study in these β-arrestin knockout mice. Here we standardized a radioligand binding methodology to characterize and quantitate βAR subtype distribution in the airway smooth muscle of wild-type C57BL/6J and β-arrestin-1 and β-arrestin-2 knockout mice. Using complementary competition and single-point saturation binding assays we found that β_2_ARs predominate over β_1_ARs in the whole lung and epithelium-denuded tracheobronchial smooth muscle of C57BL/6J mice. Quantification of βAR subtypes in β-arrestin-1 and β-arrestin-2 knockout mouse lung and epithelium-denuded tracheobronchial tissue showed that, similar to the C57BL/6J mice, both knockouts display a predominance of β_2_AR expression. These data provide further evidence that β_2_ARs are expressed in greater abundance than β_1_ARs in the tracheobronchial smooth muscle and that loss of either β-arrestin does not significantly affect the expression or relative proportions of βAR subtypes. As β-arrestins are known to modulate β_2_AR function, our analysis of βAR subtype expression in β-arrestin knockout mice airways sets a reference point for future studies exploiting these knockout mice in various disease models including asthma.

## Introduction

Bronchoconstriction is one of the salient features of asthma which is reversible by agonist-mediated activation of the β_2_ adrenergic receptor (β_2_AR), a prototypical G protein-coupled receptor (GPCR). In addition to bronchodilation, β_2_ARs also mediate bronchoprotection in asthmatic airways [1]. By virtue of these properties β_2_AR agonists remain the primary line of therapy to treat asthmatic bronchospasm.

In humans, agonist activation of β_2_ARs leads to airway smooth muscle (ASM) relaxation through activation of Gαs, cAMP accumulation and activation of protein kinase A (PKA) [2]. The distribution of βAR subtypes in human airways supports the notion that β_2_ARs mediate bronchorelaxation. Specifically, the distribution of β_1_AR and β_2_AR in human lung was reported to be 30:70 [3]; however, β_1_ARs were not detected in human bronchus [4]. βARs of human ASM and airway epithelium are known to be entirely of the β_2_ subtype [5]. βAR distribution (β_1_AR:β_2_AR) has also been studied in the airways of other animals such as pig (28:72), guinea pig (15:85), horse (26–20:74–80), dog (23:77) and rat (15:85) [6–13].

Given that *mus musculus* is one of the most commonly used species for allergic asthma models, a clear understanding of how murine airway βAR subtype expression compares to that of humans is essential to the interpretation of translational studies examining bronchodilation. Similar to that of humans, the distribution of murine βAR subtypes is heterogeneous in various tissues including lung [14, 15]. βAR expression has been studied in mouse tracheal epithelial and ASM cells. Henry *et al* reported more β_2_AR than β_1_AR expression in mouse tracheal epithelium (71% β_2_AR) but more β_1_AR than β_2_AR in ASM (69% β_1_AR) and that mouse isolated tracheal smooth muscle relaxations were mediated by β_1_AR [16, 17]. However, as in humans, airways distal to the trachea play a predominant role in determining airway resistance and recent functional data show that bronchial smooth muscle β_2_ARs play an important role in mediating bronchorelaxation in mice [15]. However, quantitative receptor expression data from murine airways is sparse in the asthma literature.

Because many asthma studies use genetically altered murine strains, interpretation of β-agonist effects on bronchoprotection and bronchorelaxation must also consider the effect of those genetic alterations on β_2_AR expression levels. Although measurement of total βAR expression is informative, changes in β_2_AR expression may be counterbalanced by changes in β_1_AR expression. This is particularly relevant given the recent use of β-arrestin knockout (KO) mice to study asthma. β-arrestins are so named because the β_2_AR was the first receptor substrate for which they were shown to terminate or “arrest” G protein-dependent cell signaling [18]. β-arrestin KO mice are a valuable tool for asthma research since loss of β-arrestin-1 expression has been shown to reduce airway bronchoconstriction (manuscript in preparation) while loss of β-arrestin-2 expression results in enhanced beta-agonist-mediated bronchorelaxation [19] and significant protection from development of the asthma phenotype [20]. However, interpretation of airway hyperresponsiveness (AHR) and bronchodilation data in these mice must take into consideration the absence of β-arrestins, not only because β-arrestins modulate airway bronchoconstriction and bronchorelaxation, but also because genetic deletion of β-arrestins may affect the expression of βARs, especially in the airways. Thus, a detailed knowledge of βAR subtype expression in β-arrestin KO mice is required for complete interpretation of AHR data. Here we standardized a radioligand binding methodology to determine if the genetic deletion of β-arrestin proteins has any impact on βAR expression in murine whole lung. Specifically, we used complementary competition and saturation binding assays to quantify βAR subtype distribution in the lung and epithelia-denuded ASM of wild-type C57BL/6J and β-arrestin-1 KO and β-arrestin-2 KO mice.

## Methods

### Ethics Statement

All animal experiments were approved by the Institutional Animal Care and Use Committee at Duke University Medical Center and were performed in accordance with the standards established by the US Animal Welfare Acts.

### Tissue preparation

Naïve mice (8–16 weeks of age) were euthanized in a CO_2_ chamber and exsanguinated. The thorax was cut open, trachea, bronchi and whole lung were quickly removed and freed of surrounding tissue. The tracheal and bronchial epithelium was denuded under microscope using Cotton tipped applicators (Puritan Medical Products Co., LLC, Maine, USA). Separated lung and trachea-bronchi were immediately placed in homogenization buffer (25 mM Tris HCl, pH 7.4, 5 mM EDTA, pH 8) with protease inhibitors (1 mM Phenyl methyl sulfonyl fluoride, 5 μg/ml Leupeptin, 10 μg/ml Benzamidine, 0.7 μg/ml Pepstatin and 10 μg/ml Aprotinin) on ice. After homogenization, unwanted cell and tissue debris were removed from the tissue lysate by a slow-speed centrifugation at 200 *g* for 10 min at 4°C and the supernatant was centrifuged at 38000 *g* for 20 min at 4°C to get membrane pellets. The pellet obtained was resuspended in ice-cold binding buffer (75 mM Tris HCl, pH 7.4, 2 mM EDTA, pH 8, 12.5 mM MgCl_2_) with protease inhibitors (5 μg/ml Leupeptin, 10 μg/ml Benzamidine, 0.7 μg/ml Pepstatin and 10 μg/ml Aprotinin) and kept frozen at -80°C until binding assay. Protein concentration was measured using Bradford reagent.

### Competition radioligand binding assays

**Fig 1:**
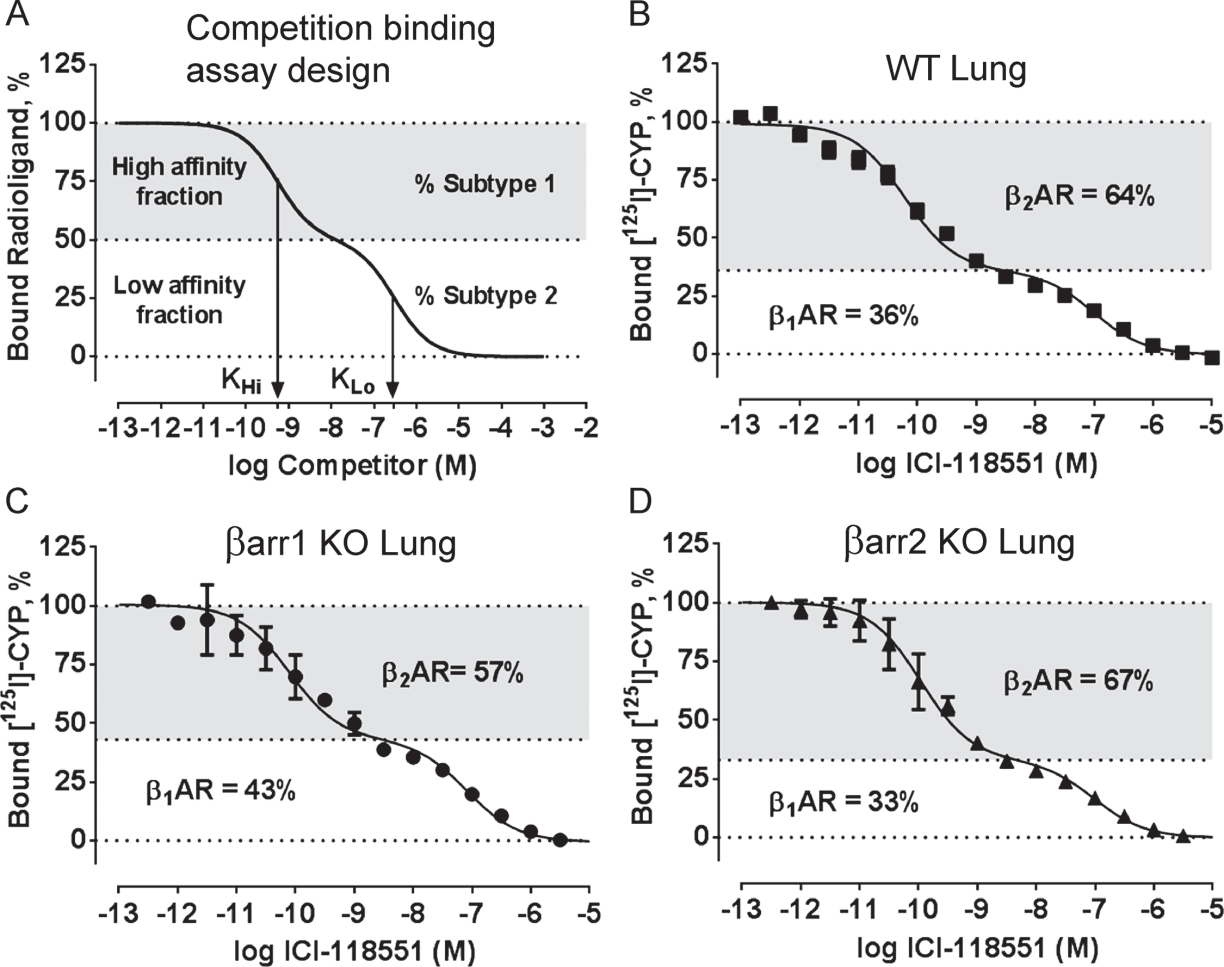
Study design of (*A*) competition radioligand binding assay to quantify β adrenergic receptor (AR) subtypes in whole lung of (*B)* wild-type C57BL/6J (WT), *(C)* β-arrestin-1 knockout (βarr1 KO), and *(D)* β-arrestin-2 knockout (βarr2 KO) mice. ***A***. Competitive displacement of a non-selective antagonist radioligand from a mixed population of receptors (50:50) by a subtype-selective competitor was simulated. Data were generated by fitting affinities of the antagonist ICI-118551 for the β_2_AR (Log Kd = -9.26) and the β_1_AR (Log Kd = -6.52) [23] to a two-site competitive binding model in GraphPad Prism. Due to its >500-fold selectivity for the β_2_AR, ICI-118551 displaces radioligand from β_2_ARs at low concentrations and from β_1_ARs at high concentrations to produce a biphasic inhibition curve. The deconvolution of high and low affinity states quantifies the fraction of each receptor subtype. In the case of ICI-118551, subtype 1 represents the β_2_AR and subtype 2 represents the β_1_AR. ***B-D***. Competition binding between (^125^I)-CYP (60 pM) and ICI-118551 (0.3 pM to 10 μM) detected 36% β_1_AR and 64% β_2_AR in WT mouse whole lung ***(B)***, 43% β_1_AR and 57% β_2_AR in βarr1 KO whole lung ***(C)***, and 33% β_1_AR and 67% β_2_AR in βarr2 KO whole lung ***(D)***. Binding parameters can be found in Table 2, with the data representing the mean ± SEM of 3–6 independent experiments performed in duplicate.

**Fig 2:**
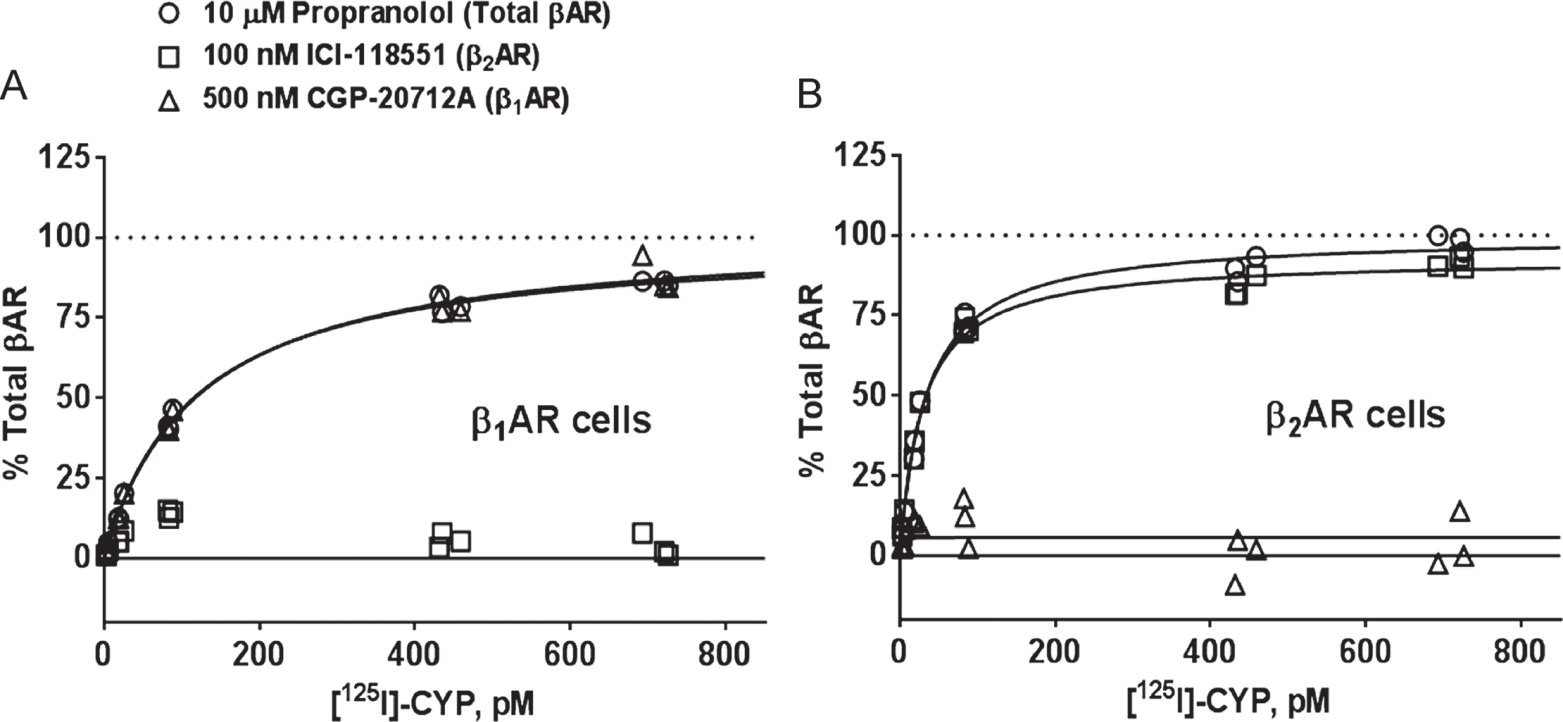
Quantification of β adrenergic receptor (AR) subtypes from a mixed population of βARs using calibrated concentrations of the β_1_AR-selective antagonist CGP-20712A and the β_2_AR-selective antagonist ICI-118551. ***A***. Proof-of-concept saturation experiments with β_1_AR-overexpressing membranes demonstrate that 500 nM CGP-20712A completely displaces (^125^I)-CYP from all available β_1_ARs, whereas 100 nM ICI-11855 is sufficiently low to not detect the β_1_AR. Total β_1_AR was set to 100% based on the displacement of (^125^I)-CYP by 10 μM propranolol. ***B***. Proof-of-concept saturation experiments with β_2_AR-overexpressing membranes demonstrate that 100 nM ICI-118551 completely displaces (^125^I)-CYP from all available β_2_ARs, whereas 500 nM CGP-20712A is sufficiently low to not detect the β_2_AR. Total β_2_AR was set to 100% based on the displacement of (^125^I)-CYP by 10 μM propranolol. Plotted data represent the individual means of three experiments performed in duplicate. Data were fit to a one-site saturation model in GraphPad Prism.

Competition binding assays measured the proportions of βAR subtypes expressed in lung membrane preparations. Similar to previous approaches that quantified receptor subtype proportions based on the differential affinity of a cold competitor for those receptor subtypes [21, 22], we used a serial dilution of the β_2_AR-selective antagonist ICI-118551 (0.316 pM to 3.16 μM) to displace (^125^I)-CYP from the β_2_AR and β_1_AR with high and low affinity, respectively. In brief, frozen membrane samples were resuspended in ice-cold binding assay buffer (50 mM Tris HCl, pH 7.4, 2 mM EDTA, 12.5 mM MgCl_2_, 180 μg/ml ascorbic acid) to yield a final membrane amount of 1.5–8 μg (lung) and 11–80 μg (tracheobronchial smooth muscle) in binding reactions containing 60 pM (^125^I)-CYP and buffer (total binding) or ICI-118551 competitor. Non-specific binding was determined in the presence of 10 μM propranolol. Pilot assays were conducted on each membrane sample to ensure that less than 10% of the total radioligand was bound. Following a 90 min incubation at room temperature, assays were terminated via harvesting onto Whatman GF/B glass fiber filters and washing with 8 mL of cold wash buffer (50 mM Tris HCl, pH 7.4, 2 mM EDTA, 12.5 mM MgCl_2_). Bound radioactivity was measured using a Packard Cobra gamma counter (Packard). Normalized data were best fit to a two-site competition binding model in GraphPad Prism (GraphPad Inc., La Jolla, CA) to yield the percentage of β_1_AR and β_2_AR present in each sample (Fig. 1). Log Ki values were fit directly in Prism by setting (^125^I)-CYP Kd equal to 0.115 nM and 0.034 nM for the β_1_AR and β_2_AR, respectively, as determined from proof-of-concept saturation binding studies (Fig. 2).

### Saturation radioligand binding assays

A complementary approach for measuring the proportions of βAR subtypes expressed on the membranes of lung and epithelia-denuded tracheobronchial smooth muscle is the single point saturation assay. In this approach, 500 nM CGP-20712A (β_1_AR-specific) and 100 nM ICI-118551 (β_2_AR-specific) were used to displace the nonselective βAR antagonist (^125^I)-cyanopindolol ((^125^I)-CYP, Perkin Elmer, MA, USA) from the β_1_AR and β_2_AR, respectively. These concentrations were based on their reported affinity for each βAR subtype [23] and were verified to only detect the desired βAR subtype in saturation experiments on β_1_AR-overexpressing [24] and β_2_AR-overexpressing [25] cell membranes (Fig. 2). The total βAR pool (set to 100%) was determined using 10 μM propranolol. In brief, frozen membrane samples were resuspended in ice-cold binding assay buffer (50 mM Tris HCl, pH 7.4, 2 mM EDTA, 12.5 mM MgCl_2_, 180 μg/ml ascorbic acid) to yield a final membrane amount of 1.5–8 μg (lung) and 11–80 μg (tracheobronchial smooth muscle) in binding reactions containing 500 pM (^125^I)-CYP and buffer (total binding) or competitor (non-specific binding). Pilot assays were conducted on each membrane sample to ensure that less than 10% of the total radioligand was bound. Assays were incubated and terminated as described above. Bound radioactivity was measured using a Packard Cobra gamma counter (Packard). Specific binding was calculated as the difference between total and nonspecific binding and expressed as fmol/mg protein given a specific activity of 4005 cpm/fmol. Control saturation binding assays using 5–750 pM (^125^I)-CYP and 0.1–0.2 μg βAR overexpressing membranes were fit via a one-site saturation model in GraphPad Prism.

### Statistics

Data were expressed as mean ± SEM. GraphPad Prism software version 5.04 (GraphPad Software, La Jolla, CA, USA) was used for nonlinear curve fitting, regression analysis and statistical calculations. Data derived from the competition experiments were best fit by a two-site binding model as determined by F test (*p* < 0.05). One way ANOVA was used to determine significant differences between genotypes, with the significance threshold set to *p* < 0.05.

## Results

### Analysis of βAR subtype expression in whole lung of wild-type C57BL/6J, β-arrestin-1 KO, and β-arrestin-2 KO mice by ICI-118551 competition binding

Subtype-selective ligands have been previously used to quantify the relative proportions of receptors in various animal tissues [21, 22]. Here we quantified βAR subtypes (Table 1) in whole lung of wild-type C57BL/6J (Fig. 1B) and β-arrestin-deficient mice (Fig. 1C and D) by measuring the competitive displacement of the non-selective βAR antagonist (^125^I)-CYP by the β_2_AR-selective antagonist ICI-118551. We chose ICI-118551 based on its >500-fold selectivity for the β_2_AR over the β_1_AR [23], thus providing accurate deconvolution of the two βAR subtypes using a two-site competition binding model [21].

**Table 1:** β adrenergic receptor (AR) subtype levels (%) in murine lung and epithelia-denuded tracheobronchial smooth muscle.

			C57BL/6J*	β-arrestin-1 knockout*	β-arrestin-2 knockout*
Lung	Competition binding	β_2_AR	64 ± 2	57 ± 3	67 ± 3
		β_1_AR	36	43	33
Lung	Saturation binding	β_2_AR	67 ± 2	63 ± 2	61 ± 2
		β_1_AR	32 ± 3	38 ± 1	32 ± 0.5
Epithelia-denuded tracheobronchial smooth muscle	Saturation binding	β_2_ AR	64 ± 3	60 ± 4	65 ± 2
		β_1_ AR	12 ± 5	13 ± 4	14 ± 4

*% βAR subtypes are expressed as mean ± SEM (n = 3). There was no effect of genotype as assessed by One way ANOVA, *p* < 0.05.

Consistent with a heterogeneous population of βAR subtypes [14, 15], ICI-118551 competition curves from whole lung membranes of wild-type mice were shallow and best fit by a two-site binding model comprising high affinity for the β_2_AR (pKi = 10.69 ± 0.06) and low affinity for the β_1_AR (pKi = 7.21 ± 0.11) (Fig. 1B) (Table 2). Calculating the fraction of receptors in each affinity state revealed that wild-type mouse lung contains 36% β_1_AR and 64% β_2_AR (Table 1).

**Table 2:** The affinities of ICI-118551 at β_2_ adrenergic receptor (AR) (pK_Hi_) and β_1_AR (pK_Lo_) in murine lung and expression values (fmol/mg) of βAR subtypes in lung and epithelia-denuded tracheobronchial smooth muscle.

			Wild-type C57BL/6J*	β-arrestin-1 knockout*	β-arrestin-2 knockout*
Lung	Competition binding	pK_Hi_	10.69 ± 0.06	10.53 ± 0.11	10.40 ± 0.07
		pK_Lo_	7.21 ± 0.11	7.29 ± 0.15	7.20 ± 0.14
Lung	Saturation binding	β_2_AR	598 ± 130	663 ± 123	737 ± 156
		β_1_AR	292 ± 79	415 ± 90	395 ± 91
Epithelia-denuded tracheobronchial smooth muscle	Saturation binding	β_2_AR	133 ± 18	126 ± 29	164 ± 48
		β_1_AR	28 ± 13	33 ± 14	42 ± 25

* Values are expressed as mean ± SEM (n = 3). There was no effect of genotype as assessed by One way ANOVA, *p* < 0.05.

We next quantified β_1_AR and β_2_AR expression in whole lung of β-arrestin-1 KO and β-arrestin-2 KO mice to determine if genetic deletion of β-arrestin alters receptor expression. In β-arrestin-1 KO mouse whole lung we found that the relative proportions of β_1_AR (43%) and β_2_AR (57%) and ICI-118551 affinities for each subtype were comparable to wild-type mice (Fig. 1C and Tables 1 and 2). Similar proportions of β_1_AR (33%) and β_2_AR (67%) and ICI-118551 affinities were detected in whole lung from β-arrestin-2 KO mice (Fig. 1D and Table 1). Small variations in βAR subtypes indicated that individual deletion of β-arrestins in the mouse lung does not alter antagonist binding or the proportion of βAR subtypes.

Competition binding assays were not performed on the tracheobronchial tissue of wild-type and β-arrestin deficient mice given the limitations associated with collecting large amounts of tracheobronchial tissue. Single-point saturation assays were used for this tissue as reported below.

### Analysis of βAR subtype expression in whole lung of wild-type C57BL/6J, β-arrestin-1 KO, and β-arrestin-2 KO mice by single-point saturation

We developed a single-point saturation binding assay to quickly and efficiently calculate receptor density (B_max_). Specifically, empirically-determined concentrations (Fig. 2) of CGP-20712A (a β_1_AR selective antagonist) and ICI-118551 (a β_2_AR selective antagonist) were used to displace a saturating concentration of the non-selective βAR antagonist (^125^I)-CYP (500 pM) in a subtype-selective manner from a pool of βARs. A saturating concentration of the non-selective antagonist propranolol (10 μM) was used to determine the total pool of βARs (set to 100%) in each membrane sample. Proof-of-concept experiments in which 10 μM propranolol was used to detect the maximal amount of each βAR subtype in overexpressing cell lines revealed that 500 nM CGP-20712A was sufficiently low to occupy all β_1_ARs (Fig. 2A) but not detect the β_2_AR (Fig. 2B). Similarly, 100 nM ICI-118551 was sufficiently low to occupy all β_2_ARs (Fig. 2A) but not detect the β_1_AR (Fig. 2B). These concentrations were also consistent with calculations of fractional occupancy for competitive binding between two ligands [26].

**Fig 3:**
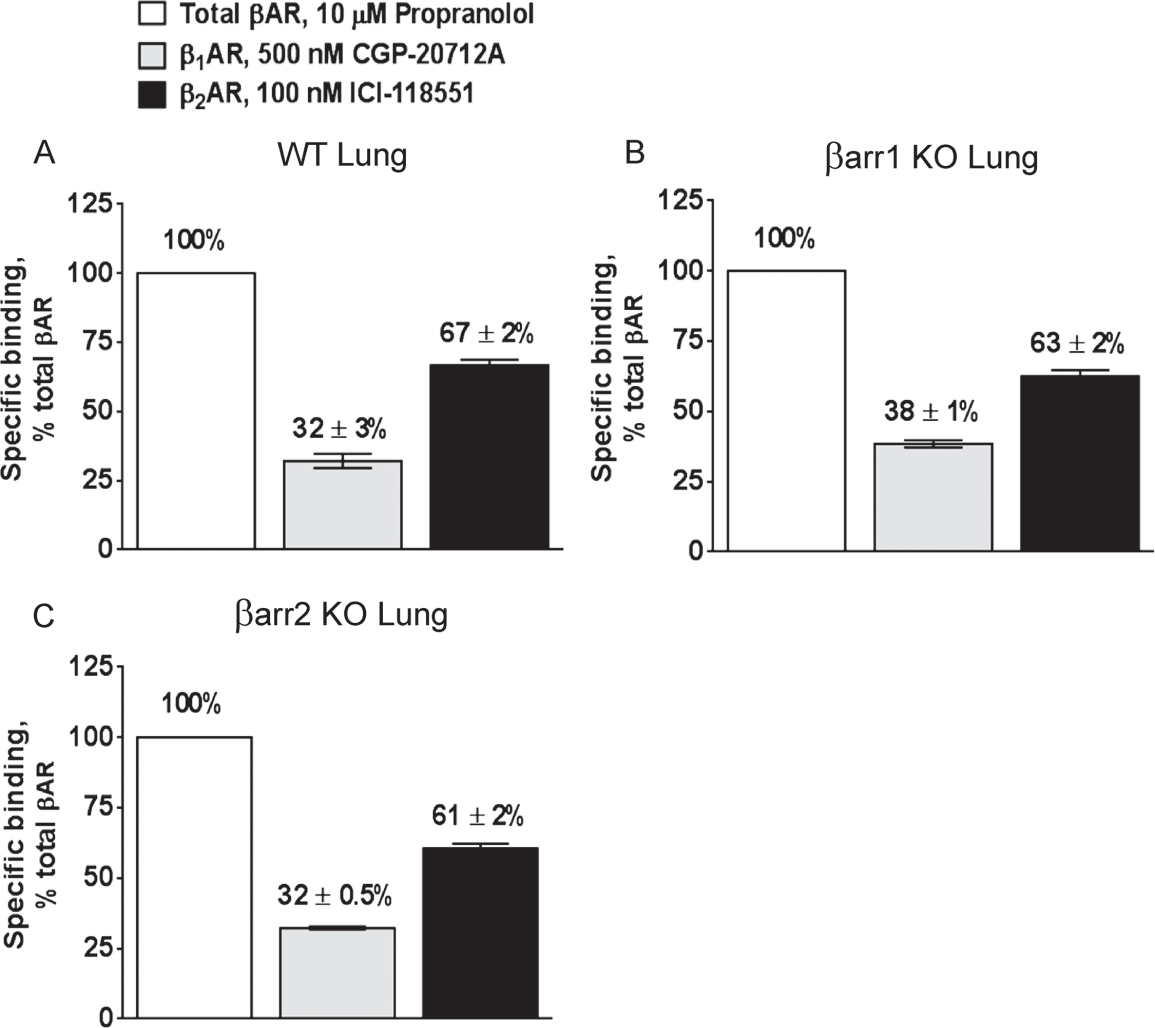
Estimation of β adrenergic receptor (AR) subtypes by single-point saturation binding assay in whole lung of (*A*) wild-type (WT) C57BL/6J, (*B*) β-arrestin-1 knockout (βarr1 KO), and (*C*) β-arrestin-2 knockout (βarr2 KO) mice. The competitive displacement of the non-selective radiolabeled antagonist (^125^I)-cyanopindalol (CYP) (500 pM) by 500 nM CGP-20712A and 100 nM ICI-118551 quantifies the proportions of β_1_AR and β_2_AR, respectively. Propranolol, a nonselective βAR blocker, gives a measure of total βAR present in each tissue. ***A***. WT: β_1_AR = 32 ± 3%; β_2_AR = 67 ± 2%; 100% corresponds to 887.2 ± 168 fmol/mg. ***B***. βarr1 KO: β_1_AR = 38 ± 1%; β_2_AR = 63 ± 2%; 100% corresponds to 1072 ± 222 fmol/mg. ***C***. βarr2 KO: β_1_AR = 32 ± 0.5%; β_2_AR = 61 ± 2%; 100% corresponds to 1221 ± 277 fmol/mg. Data represent the mean ± SEM of 3 independent experiments performed in quadruplicate.

When used to assess the proportion of βAR subtypes in the whole lung of wild-type C57BL/6J mice, single-point saturation experiments yielded 32% β_1_AR and 67% β_2_AR (Fig. 3A and Table 1). This was in good agreement with the proportion of βAR determined from ICI-118551 competition experiments (Fig. 1B). The proportions of β_1_AR and β_2_AR in whole lung of β-arrestin-1 KO (38% and 63%, respectively) (Fig. 3B) and β-arrestin-2 KO mice (32% and 61%, respectively) (Fig. 3C) were also comparable to the results from competition experiments, showing *1)* no effect of genotype on βAR subtype expression and *2)* that the two approaches yielded equivalent information. Additionally, as shown in Table 2, the total βAR receptor density in the lung of C57BL/6J mice (598 ± 130 and 292 ± 79 fmol/mg for β_2_AR and β_1_AR, respectively) was in good agreement with total βAR expression measured in our prior study (1004 ± 54 fmol/mg) [15].

### Analysis of βAR subtype expression in epithelia-denuded tracheobronchial smooth muscle of wild-type C57BL/6J, β-arrestin-1 KO, and β-arrestin-2 KO mice by single-point saturation

**Fig 4:**
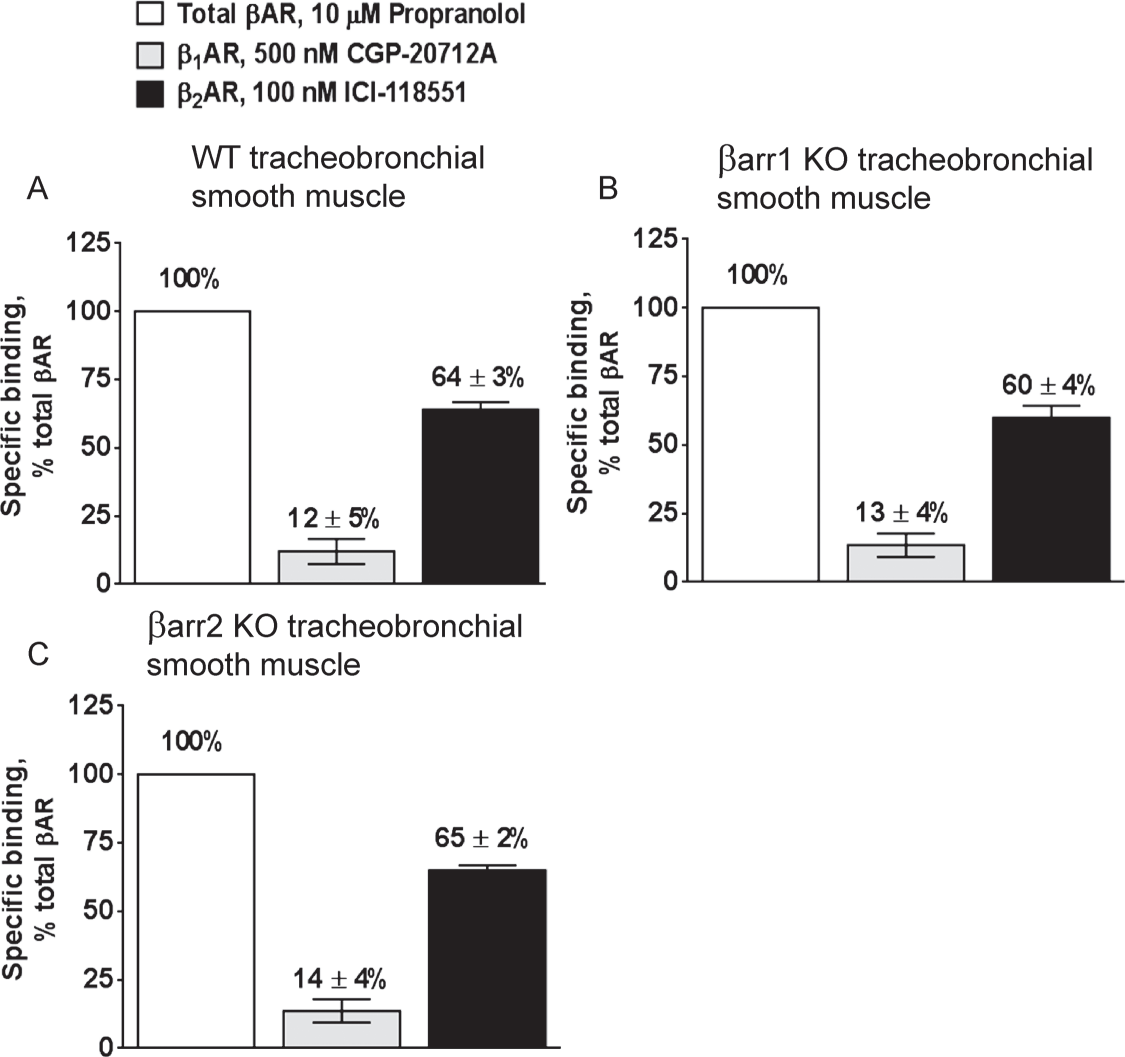
Estimation of β adrenergic receptor (AR) subtypes by single-point saturation binding assay in tracheobronchial smooth muscle of (*A*) wild-type (WT) C57BL/6J, (*B*) β-arrestin-1 knockout (βarr1 KO), and (*C*) β-arrestin-2 knockout (βarr2 KO) mice. The competitive displacement of the non-selective radiolabeled antagonist **(** ^125^I)-cyanopindalol (CYP) (500 pM) by 500 nM CGP-20712A and 100 nM ICI-118551 quantifies the proportions of β_1_AR and β_2_AR, respectively. Propranolol, a nonselective βAR blocker, gives a measure of total βAR present in each tissue. ***A***. WT: β_1_AR = 12 ± 5%; β_2_AR = 64 ± 3%; 100% corresponds to 208.2 ± 28 fmol/mg. ***B***. βarr1 KO: β_1_AR = 13 ± 4%; β_2_AR = 60 ± 4%; 100% corresponds to 213 ± 55 fmol/mg. ***C***. βarr2 KO: β_1_AR = 14 ± 4%; β_2_AR = 65 ± 2%; 100% corresponds to 255.7 ± 82 fmol/mg. Data represent the mean ± SEM of 3 independent experiments performed in quadruplicate.

We next quantified βAR subtypes in the tracheobronchial tissue of mice given that bronchial smooth muscle β_2_ARs mediate bronchorelaxation in mice [15]. Using single-point saturation analysis we determined for the first time that epithelia-denuded tracheobronchial smooth muscle of wild-type C57BL/6J mice contains 12% β_1_AR and 64% β_2_AR (Fig. 4A). Similar levels of expression were observed in β-arrestin-1 KO mice (13% β_1_AR and 60% β_2_AR; Fig. 4B) and β-arrestin-2 KO mice (14% β_1_AR and 65% β_2_AR; Fig. 4C) (Table 1).

## Discussion

Radioligand binding assays are extremely powerful tools to study receptor expression and subtype proportion under normal and disease states and during administration of drug therapies. Binding methods exploit the basic principle of competitive binding between nonselective radioligands and selective cold ligands to quantitate the proportion of receptor subtypes [21, 22, 27]. Here we standardized an approach using complementary competition and saturation binding assays to evaluate the βAR subtype distribution in murine wild-type and β-arrestin KO whole lung. Consistently, we found comparable receptor density results between ICI-118551 competition and single-point saturation assays. Thus, the single-point saturation assay can be used to quantify βAR subtypes, the main advantage being reduction in the amount of tissue sample required for each assay. This permits radioligand binding analysis with fewer animals and improved cost effectiveness. Our careful validation of subtype-specific ligand concentrations and assay conditions, both of which are critical to the success of radioligand binding experiments, is an advance over previous reports in which βAR subtypes were measured. Our work may also provide a partial explanation for the discrepancy in murine airway distribution of βAR subtypes reported in the literature [16, 17] and below we provide further comment on these differences. Furthermore, we denuded the tracheal and bronchial epithelium and used only the smooth muscle for membrane preparation since mouse tracheal epithelium expresses a higher density of β_2_AR than ASM [17]. The airways distal to the trachea influence the airway resistance during bronchoconstriction [15] and ASM is an important effector cell type involved in AHR, the cardinal feature of asthma [28, 29]. By carefully denuding the epithelium, we were able to focus our analysis on the receptors expressed in the smooth muscle and thus provide correct reference values of βAR subtypes involved in bronchodilation in these β-arrestin KO mice.

We report that the β_2_AR subtype was predominantly expressed in whole lung and epithelium-denuded tracheobronchial smooth muscle membranes of C57BL/6J and β-arrestin-1 KO and β-arrestin-2 KO mice. The data provide evidence that β_2_ARs are expressed in greater abundance than β_1_ARs in the tracheal smooth muscle. Until recently, the general assumption was that mouse airways use β_1_ARs to relax and this was primarily based on the receptor expression studies by Henry and colleagues who showed that β_1_AR expression predominates [16, 17]. Several reasons may account for the discrepant results. First, in our study, the tracheal and bronchial epithelium was separated from the smooth muscle, but this was not the case in the Henry and colleague experiments [16, 17]. By carefully removing epithelial cells we were able to unambiguously quantify the proportion of βAR subtypes in the airway smooth muscle extending to the bronchi. Secondly, we used cell membranes for direct binding studies whereas Henry and colleagues used quantitative autoradiography.

The large excess of β_2_AR over β_1_AR expression found in the current study is supportive of results from our prior study which showed that β-agonist-mediated bronchorelaxation does not occur in β_2_AR-KO mice [15]. Taken together, these results point to an important role for β_2_ARs, not β_1_ARs, in mediating murine bronchorelaxation [15]. Collectively, the murine β_2_AR expression and functional evidence vouches for the suitability of this species as a model to study the effects of β-agonists in human allergic asthma.

Agonist-mediated activation of the β_2_AR is responsible for the reversal of bronchoconstriction in human and murine asthma. Paradoxically, expression and activation of β_2_ARs is required for development and pathogenesis of the asthma phenotype in mice and worsening of the asthma phenotype in humans [30–32]. Increasing evidence suggests that separate G protein- and β-arrestin-dependent signaling pathways downstream of β_2_AR are responsible for these paradoxical effects of β_2_AR activation in asthma [20, 32, 33]. We previously showed that β-arrestin-2 deletion protects mice against allergen-induced asthma [20] and enhances β_2_AR-induced relaxation in murine ASM [19]. Thus, therapeutic strategies that inhibit β-arrestin-2 functions or bias β_2_AR signaling toward the Gs/cAMP, or away from the β-arrestin-mediated, signaling pathway may be beneficial in asthma [34, 35]. In this context, β-arrestin KO mice represent valuable investigative tools in our lab and elsewhere for the study of asthma; however, measuring βAR subtype expression density in the lung and airways is essential to the full interpretation of asthma phenotypes. Herein, we are the first to show that genetic deletion of either β-arrestin-1 or -2 does not affect the expression of lung or tracheobronchial β_1_- or β_2_-ARs in naive mice. Going forward it will be important to understand how various models of allergen exposure may impact the expression of lung and airway βARs and β-arrestins. For example, we previously showed that allergen (OVA) sensitization and chronic allergen challenge leads to a significant reduction in whole lung expression of total βARs (from 1004 ± 54 fmol/mg to 598 ± 88 fmol/mg) [15] and an elevation in whole lung expression of β-arrestin-2 (under second review Chen *et al.*, Am J Respir Cell Mol Biol—“Genetic deletion of β-arrestin-2 mitigates established airway hyperresponsiveness in a murine asthma model”). Utilization of the methods described herein will facilitate the measurement of βAR subtype expression, especially in tissues that are limited in size, thus providing information needed for correct interpretation of lung mechanics data in a variety of murine models of asthma.

In summary, our study provides the first detailed reference levels of lung and airway βAR subtype densities in β-arrestin-1 KO and β-arrestin-2 KO mice. Our data substantiate the notion that β_2_ARs mediate murine bronchial smooth muscle bronchorelaxation. Our study also demonstrates that genetic deletion of β-arrestin-1 or -2 does not significantly alter the expression of β_1_ – or β_2_-ARs in naïve whole lung or tracheobronchial airway smooth muscle cells.
